# Residual low-frequency hearing after early device activation in cochlear implantation

**DOI:** 10.1007/s00405-023-07887-0

**Published:** 2023-03-21

**Authors:** Stefanie Bruschke, Uwe Baumann, Timo Stöver

**Affiliations:** Goethe University Frankfurt, University Hospital, ENT Department, Theodor-Stern-Kai 7, 60590 Frankfurt a. M, Germany

**Keywords:** Cochlear implant, Early fitting, Residual hearing, Speech recognition

## Abstract

**Purpose:**

The cochlear implant (CI) is a standard treatment for patients with severe to profound hearing loss. In recent years, early device activation of the sound processor after 2–3 days following surgery has been established. The aim of this study is to evaluate the residual hearing of CI patients with substantial preoperative low-frequency hearing after early device activation over a period of 12 months.

**Methods:**

Results were compared between an early fitted group (EF) with device activation to less than 15 days after CI surgery and a control group (CG) with device activation after 3–6 weeks. In total, 57 patients were divided into EF group (*n* = 32), and CG (*n* = 25). Low-frequency residual hearing and speech recognition in quiet and in noise were compared over an observation period of 12 months.

**Results:**

No significant difference (*p* > 0.05) in the residual low-frequency hearing PTA_low_ between EF and CG was found, neither preoperatively (EF 33.2 dB HL/CG 35.0 dB HL), nor postoperatively (EF 46.8 dB HL/CG 46.2 dB HL). In both groups, postoperative residual hearing decreased compared to preoperative and remained stable within the first year after CI surgery. Furthermore, both groups showed no significant differences (*p* > 0.05) in speech recognition in quiet and in noise within the first year.

**Conclusion:**

Early device activation is feasible in CI patients with preoperative low-frequency residual hearing, without an additional effect on postoperative hearing preservation.

## Introduction

The cochlear implant (CI) is a standard treatment for patients with severe to profound hearing loss [1, 2]. The usage of a CI improves the speech recognition [3] in everyday life as well as the quality of life [4]. Since the introduction of the CI, the indication criteria have been expanded. Initially only completely deaf patients were treated with a cochlear implant [5], later also patients with low-frequency residual hearing [6]. Also the treatment procedure has changed over time. Until a few years ago, the sound processor was initially activated after a standard healing phase of 3–6 weeks [1, 2, 7, 8], to ensure that the wound healing process was completed. With advances in surgical technique and instrumentation, as well as the use of the small incision technique [9], the wound is smaller and heals faster, with less postoperative pain [10] and wound swelling [11]. This allows earlier initial sound processor activation within only 2–3 days after surgery. This significantly shortens the time period between surgery and first fitting [12], thus hearing rehabilitation may start much sooner. Previously, we demonstrated the feasibility and safety of early processor activation after CI surgery also after long-term follow-up [12, 13]. As in other studies [3, 14, 15] it was shown that the early fitting of the sound processor is a safe and effective procedure with no known additional medical risks or complications. Facilitating early fitting, comparable speech recognition in quiet was achieved compared to standard fitting [12, 13]. Despite these advantages, until now it is unknown if early sound processor activation may influence residual hearing in patients following cochlear implantation. Hearing preservation today is a general aim in CI surgery. For postoperative hearing preservation, an atraumatic surgical technique for structure preservation is required [5, 16, 17]. Helbig et al. [16] presented data on long-term acoustic hearing preservation after CI und concluded that preservation is feasible in patients fitted 4–6 weeks after implantation (standard healing phase).

The advantage of using a hearing preservation technique for CI surgery [18–20] is that the acoustic low-frequency residual hearing can be used for electric-acoustic stimulation (EAS). It is a combination of acoustic stimulation via hearing aid function in the apical area of the cochlear and electrical stimulation of the basal parts of the cochlear [5, 21]. The use of low-frequency residual hearing in EAS patients leads to an improvement in speech recognition [22]. The EAS/hybrid usage of the sound processor has become established in patients with severe high-frequency hearing loss [5, 20].

The investigation of hearing preservation associated with the early initial processor activation is of interest because it is not clear whether early electrical stimulation has an effect on residual acoustic hearing function.

Intracochlear healing processes are completed after a period of 4–5 weeks [2]. It remains unclear, whether acoustic and electrical stimulation after traumatic electrode insertion could lead to apoptosis of the hair cells and thus to the loss of residual hearing. The insertion of the electrode is potentially traumatic for the sensitive hearing organ [23]. Therefore, it is unknown whether early processor activation affects hair cell function and whether the immune response induced by electrode insertion [24], in combination with early electrical stimulation, may deteriorate hearing thresholds in the long term. In case of other traumatic events, such as noise trauma or acute sudden hearing loss, acoustic overstimulation with high stimulation levels is avoided. For example, the German audiological association ADANO recommends avoiding examinations such as BERA (brainstem evoked response audiometry), CERA (cortical evoked response audiometry), stapedius reflex and electrocochleography with high sound pressure levels within eight days after sudden hearing loss [25].

The aim of this study therefore was to examine whether early fitting of the sound processor has an impact on the postoperative low-frequency residual hearing as a potential negative side effect following the early fitting procedure. Therefore, long term data on residual hearing loss and of speech recognition in quiet and in noise were compared over a period of 12 months between patients who received an early device activation (study group) and patients whose processor was initially activated after the standard healing phase (control group). It is assumed that long term preservation of the residual hearing is possible after early initial processor activation.

To our knowledge, there are currently no studies that have examined the postoperative residual hearing preservation after CI surgery in association with the early sound processor activation within a long term follow-up of 12 months.

## Materials and methods

**Fig. 1:**
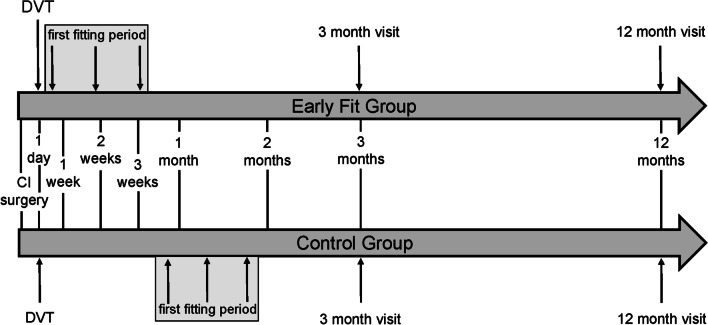
Time schedule for post-surgery care within 12 months after CI surgery for early fitting group (EF, *above*) and control group (CG, *below*). *DVT* digital volume tomography, *CI* cochlear implant

A total of 57 patients were enrolled in the prospective study. The early fitting group (EF) included 32 patients (18 males, 14 females). In this group, the sound processor was initially activated within a maximum of 15 days after CI surgery. In the control group (CG), which included 25 patients (8 males, 17 females) the processor was first activated after a standard healing phase of 3–6 weeks. The CI surgery was carried out in approximately the same time span in both patient groups. All patients had a minimum age of 18 years and fulfilled the inclusion criteria for an EAS/hybrid CI surgery. Therefore, a sufficient residual low-frequency hearing was necessary. The criterion was set at a maximum hearing loss of 70 dB HL at a test frequency of 500 Hz. The assignment to the respective study group (EF or CG) was non-randomized and based on the willingness of the patients to participate in the early fitting process. Patients who did not want an early processor activation were assigned to the CG. The demographic data of the patients are shown in Table 1. In both study groups, the “small incision” [9] technique was applied for CI surgery. A DVT (digital volume tomography) was performed postoperatively in all patients to confirm the placement and position of the electrode. The first fitting interval with initial device activation was accomplished in three fitting appointments. After initial device activation the two additional fitting appointments during the first fitting interval took place within two weeks. In both groups, regular follow-up visits were carried out after 3, 6 and 12 months following initial processor activation. In all clinic appointments, the wound healing status was assessed by a physician, the sound processor was programmed by an audiologist and audiometric assessments were performed. For data analysis, the air conduction thresholds were regarded, which were measured via headphones (Telephonics TDH-39P, Farmingdale, NY, United States). To assess low-frequency residual hearing, the PTA_low_ (pure tone average) was calculated as the average hearing loss at 125 Hz, 250 Hz and 500 Hz. Furthermore, speech recognition in quiet was measured using the Freiburg multisyllabic and monosyllabic word test at 65 dB SPL presentation level in free field condition. In addition, the 50% speech reception threshold (SRT) of the adaptive Oldenburg Sentence Test (Oldenburger Satztest, OlSa) [26] in quiet was determined in free field at a starting level of 55 dB SPL. To assess speech recognition in noise, the OlSa was carried out with adaptive noise level (olnoise signal) [26] in free field condition. A fixed speech level of 65 dB SPL and a noise starting level of 60 dB SPL was used to determine SRT. Speech and noise signal were presented from the front (S_0_N_0_). Both OlSa tests were performed in a closed setup. The study data were collected preoperatively, at the third day of the first fitting interval and at the follow-up intervals after 3 and 12 months following CI surgery. The study procedure is shown schematically in Fig. 1.

**Table 1:** Demographical data of early fitting group and control group

	Early fitting group	Control group
Cases, *n*	32	25
Age		
Mean, years	58.1	59.9
Min/Max, years	21/83	35/85
Gender	18 male, 14 female	8 male, 17 female
Manufacturer		
Advanced Bionics	2	–
Cochlear	19	12
MED-EL	11	13
Device		
HiRes Ultra SlimJ	2	–
CI522	11	12
CI532	3	–
CI622	5	–
Flex20	–	1
Flex24	6	7
Flex26	2	1
Flex28	3	4
Duration of profound hearing loss		
Mean, years	17.5	27.0
Min/Max, years	1.0/44.0	5.0/65.0
Unknown, *n*	13	10
Hearing aid experience		
Mean, years	14.5	25.6
Min/Max, years	1.0/40.0	2.0/60.0
None, *n*	4	2
Unknown, *n*	4	5

### Statistical evaluation

Normal distribution of the data was tested using the Kolmogorov–Smirnov test. In case of a normal distribution, the statistical comparison of the results between the groups was performed using the t-test for unpaired samples. The data was first tested for variance equality via the Levene test. To compare the results within the groups, the *t* test for connected samples was used. For data that did not show a normal distribution, statistical comparison of the results between groups were carried out via the Mann–Whitney-*U*-Test. Within groups, comparison of the results were statistically evaluated using the Wilcoxon signed-rank test. A Bonferroni correction was used to analyze the data within the groups after the respective statistical tests had been carried out. A *p* value of less than 0.05 was considered statistically significant. Statistical analysis were performed using SPSS Statistics 24.0 (IBM Corporation, Endicott, NY, United States). To obtain valid statistical statements about certain processes over time, a complete case approach of the data was performed. This resulted in lower case numbers. However, subsequent analyzes comprising all available data showed comparable results. Therefore, for comparison between both study groups, the use of all data was prioritized to perform an analysis with larger case numbers. For data evaluation within groups, the complete case approach was applied to allow better comparability of results over time.

## Results

The early fitting procedure allowed a much earlier initial activation of the sound processor than after awaiting the standard healing phase. The EF (*n* = 32) was first fitted after 3.0 days (median) and thus significantly earlier (*U* = 1.000, *Z* = -6.451, *p* < 0.001, Mann–Whitney-*U*-Test) than the CG (*n* = 25) after 28.0 days (median). The study data were collected over a period of 12 months. Since not all patients were able to attend their follow-up appointments after 3 and 12 months following initial device activation for personal or therapeutic reasons (e.g. ongoing stationary rehabilitation), not all data sets were complete.

The demographic data (see Table 1) showed a comparable age of both experimental groups (EF 58.1/CG 59.9 years, mean). At 27 years (mean), the CG had a longer duration of profound hearing loss than the EF, which had a duration of 17.5 years. In addition, CG patients had more hearing aid experience (25.6 years) than EF patients (14.5 years). However, the duration of profound hearing loss can often not be precisely determined. The patient’s memory of the onset of the hearing loss is often vague or cannot be clearly determined because of progressive hearing loss, especially if residual hearing is still present.

Nevertheless, it is known that the duration of profound hearing loss has an impact on the performance with CI [27]. In the EF subject group 8 patients were implanted with Flex24 (6) and Flex26 (2) electrodes, in the CG subject group 9 patients with Flex20 (1), Flex24 (7) and Flex26 (1). Those are shorter and more flexible electrodes that can promote the preservation of postoperative residual hearing [28]. In patients fitted with a device manufactured by Cochlear, the straight electrode arrays CI522/CI622 (EF 11/5, CG 12) and the perimodiolar flexible CI532 (EF 3) were used. No differences in hearing preservation between CI522 and CI532 were expected. Preservation of residual hearing is feasible with both electrode arrays [29].

### Residual hearing

**Fig. 2:**
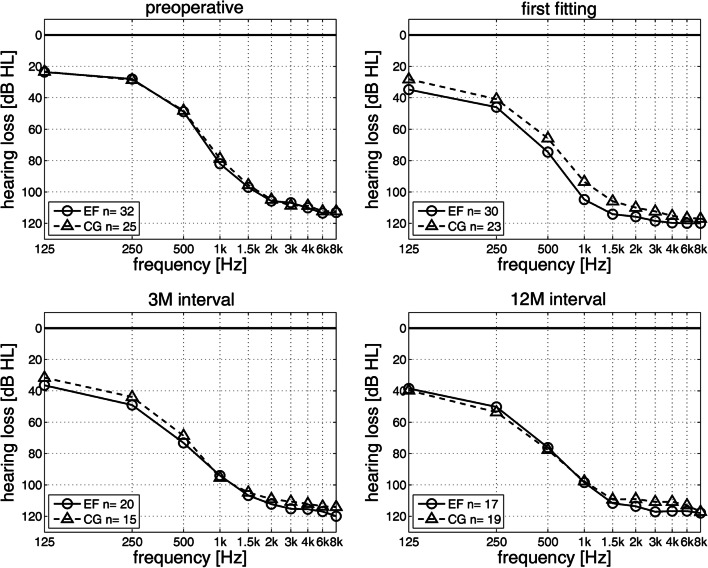
Mean pure tone thresholds for early fitting group (EF) and control group (CG). Air conduction measured via headphones. **A** preoperative data. **B** first fitting appointment. **C** 3-month interval (3 M). **D** 12-month interval (12 M). All available data

**Fig. 3:**
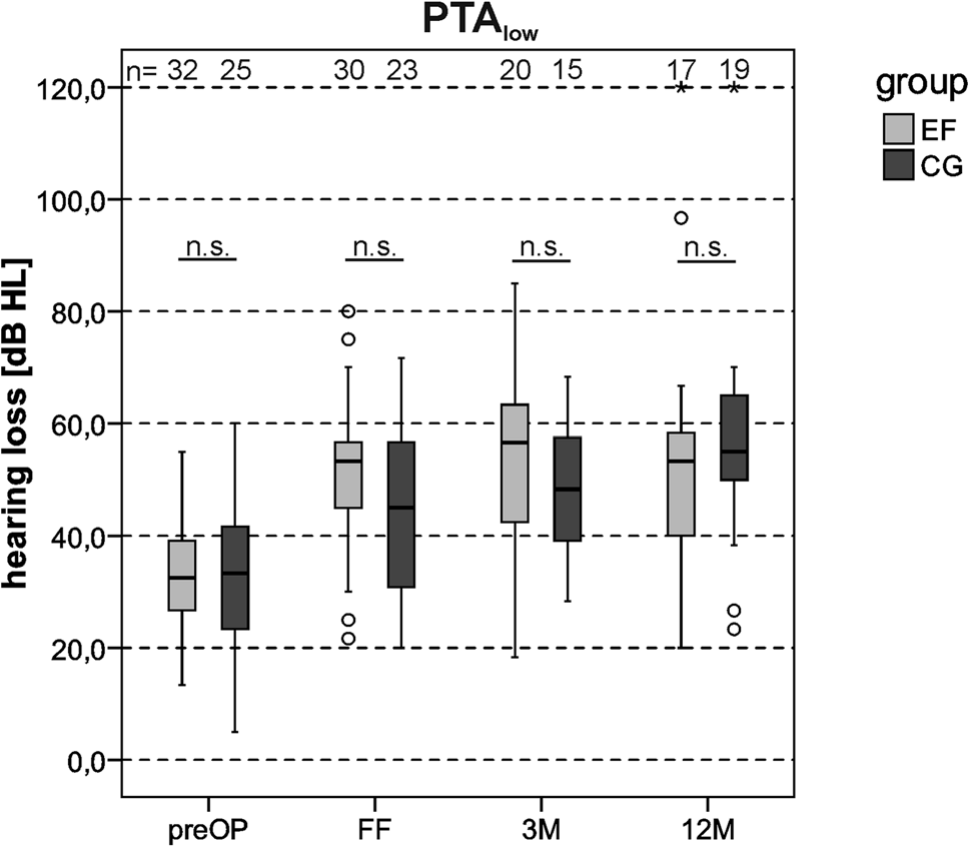
PTA_low_ (average hearing loss at 125 Hz, 250 Hz, 500 Hz) for early fitting group (EF) and control group (CG). Data collection preoperatively (preOP), at first fitting (FF), 3-month interval (3 M) and 12-month interval (12 M). Measured via headphones. All available data. *n.s.* not significant (*p* > 0.05)

To assess the preservation of residual low-frequency hearing up to 12 months after CI surgery, the pre- and postoperative results for pure tone audiometry were compared for each group (see Fig. 2). At the first fitting appointment the pure tone audiograms showed a difference between EF and CG. The EF showed a more pronounced low-frequency hearing loss compared to the CG (see Fig. 2B). In the considered frequency range up to 1 kHz no difference was found between EF and CG with preoperative data and postoperative measurements for the 3- and 12-month interval (see Fig. 2A, C, D). Furthermore, it can be observed that both EF (*n* = 18) and CG (*n* = 14) had better preoperative residual hearing than postoperative. In addition, no substantial change in residual hearing between the first fitting appointment and the follow-up intervals was observed in either group. The postoperative residual low-frequency hearing thus remained stable. In Fig. 3 the PTA_low_ for both groups over an observation period of 12 months is shown. Comparing results between groups, no significant differences (*p* > 0.05, *t* test) in PTA_low_ between EF and CG within the observation period was present (no complete case approach). Within the EF, a significant difference in PTA_low_ was found between preoperative data (33.2 dB HL) and postoperative measurements on first fitting appointment (46.8 dB HL, *t*(10) = − 3.44, *p* = 0.038, *t*-test/Bonferroni correction (BC)), 3-month (46.5 dB HL, *t*(10) = − 3.93, *p* = 0.017, *t* test/BC) and 12-month interval (54.4 dB HL, *t*(10) = -3.88, *p* = 0.018, *t* test/BC). Within the CG there was a significant difference in PTA_low_ between preoperative data (35.0 dB HL) and the results at 12-month interval (53.3 dB HL, *t*(10) = − 3.41, *p* = 0.040, *t* test/BC). Due to the small number of cases after complete case approach (EF/CG *n* = 11), however, the results of the statistical evaluation within the groups can only be interpreted to a limited extent. Complete postoperative hearing loss was recorded in one patient from the EF (5.9%, 1/17) and CG (5.3%, 1/19) at the time of the 12-month interval.

### Speech recognition

**Fig. 4:**
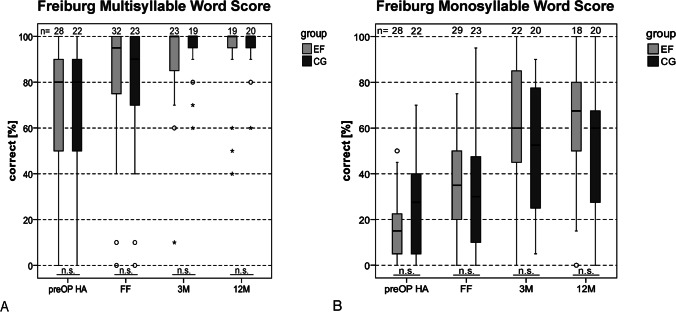
Aided speech recognition in quiet (% correct) of early fitting group (EF) and control group (CG). Preoperative data obtained with hearing aid (preOP HA), postoperative data measured with cochlear implant at first fitting (FF), 3-month interval (3 M) and 12-month interval (12 M). **A** Freiburg multisyllabic word score (numerals). **B** Freiburg monosyllabic word score. Free field presentation, 65 dB SPL presentation level. Opposite ear blocked. All available data. *n.s.* not significant (*p* > 0.05)

**Fig. 5:**
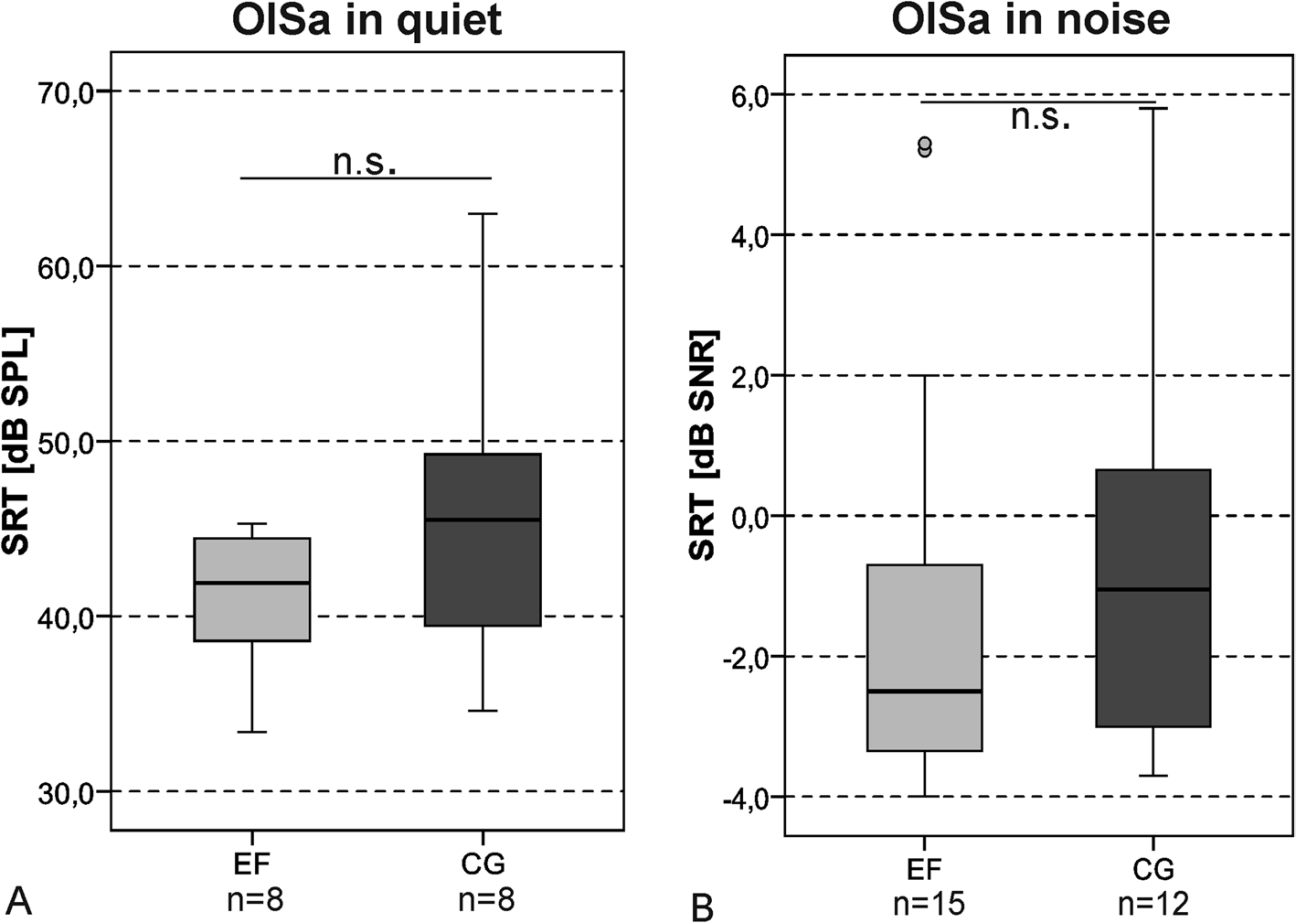
Speech reception threshold (SRT) of early fitting group (EF) and control group (CG) at 12-month interval. **A** adaptive OlSa in quiet with 55 dB SPL start level. **B** adaptive OlSa in noise (olnoise) with fixed speech level at 65 dB SPL and adaptive noise at 60 dB SPL starting level. Opposite ear blocked. All available data. *n.s.* not significant (*p* > 0.05)

When analyzing the results of the Freiburg multisyllabic word test, there was no significant difference (*p* > 0.05, Mann–Whitney-*U*-Test) between the EF and CG, neither preoperatively nor postoperatively (see Fig. 4A). The results for the Freiburg monosyllabic word test also showed no significant difference (*p* > 0.05, *t* test) in speech recognition between EF and CG, both preoperatively and postoperatively (see Fig. 4B). The evaluation of the results within the EF showed a significant improvement in postoperative word score compared to preoperative word score (13.2%) at first fitting interval (37.1%, *t*(13) = − 3.74, *p* = 0.012, *t* test/Bonferroni correction (BC)), 3-month interval (57.5%, *t*(13) = − 4.47, *p* = 0.006, *t* test/BC) and 12-month interval (60.7%, *t*(13) = − 5.43, *p* < 0.001, *t* test/BC). When evaluating the results from the CG, a significant improvement (*t*(16) = − 4.50, *p* < 0.001, *t* test/BC) in preoperative monosyllabic word score (22.3%) was found compared to the 12-month interval (53.8%). The results of the OlSa in quiet (see Fig. 5A) and the OlSa in noise (see Fig. 5B) showed that both study groups had comparable SRTs after 12 months of CI experience. The OlSa SRT in quiet (EF/CG, 41.9 dB SPL/45.5 dB SPL, median) showed no significant difference (*t*(14) = − 1.36, *p* = 0.195, *t* test) between EF and CG. Also for the OlSa SRT in noise (EF/CG, -2.5 dB SNR/− 1.1 dB SNR, median) no significant difference (*t*(25) = − 0.51, *p* = 0.613) between EF and CG was found. However, the case numbers for OlSa in quiet are low EF, *n* = 8/CG, *n* = 8), so that the evaluation of these data is of limited value.

## Discussion

As expected, initial processor activation of patients who received an early fitting procedure took place 3 days after surgery, whereas in the CG an average of 28 days was observed. Thus, the EF patients were able to gain listening experience much earlier.

### Residual hearing

#### Pure tone thresholds

In both study groups, preoperative pure tone thresholds were comparable. In the initial fitting interval, EF group subjects tended to have slightly greater postoperative hearing loss than CG subjects did. An explanation for this observation might be a temporary degradation generated by early postoperative effects. After 3–6 weeks, the postoperative healing processes are already further advanced [2], and after 12 months of CI experience, both study groups showed nearly the same residual hearing in the low-frequency range up to 1 kHz. The same degree of postoperative deterioration was observed in both study groups as in previous studies of hearing preserving CI surgery [16, 17, 30]. In both EF and CG, postoperative residual hearing remained stable within the first year after CI surgery. This has also been described in other studies that have examined residual hearing after CI surgery [6, 16, 19].

As also shown by Gautschi-Mills et al., 2019 [30], postoperative hearing preservation after CI surgery occurred in the majority of patients in both study groups. Only one case in both groups showed complete postoperative hearing loss at the 12-month interval (EF 5.9%, CG 5.3%). This is approximately the same as the 8% reported by Gautschi-Mills et al., 2019 [30]. Despite hearing preserving surgical techniques, complete postoperative hearing loss is possible in some cases, i.e., complete loss of residual hearing function [5, 31]. A possible reason could be a scala disruption of the electrode array from the scala tympani to the scala vestibule [32]. In both cases of complete postoperative loss of residual low-frequency hearing, the processor was programmed from EAS/hybrid mode to standard mode. The patients were able adapt to the altered distribution of filter bank frequencies. At the 12-month interval, both patients with complete hearing loss showed an adequate monosyllabic word score of 65% and 70%. Even after complete postoperative loss of residual hearing, patients can still achieve satisfactory results with the CI [19].

#### PTA_low_

There was no significant difference in PTA_low_ between EF and CG in all study intervals. However, comparison of PTA_low_ between preoperative and postoperative measurements showed a significant difference, both for EF and CG. In the EF, the PTA_low_ already decreased at the initial sound processor activation (from 33.2 dB HL preoperatively to 46.8 dB HL postoperatively. This deterioration showed statistical significance in the CG at the 12-month interval (from 35.0 dB HL preoperatively to 53.3 dB HL postoperatively). At the 12-month interval, both groups showed a significant decrease in residual low-frequency hearing compared to preoperative measurements. In the EF, the PTA_low_ decreased by 21.2 dB HL, in the CG the PTA_low_ decreased by 18.3 dB HL. The study by Helbig et al., 2016 [16] also examined the residual low-frequency hearing of EAS patients, where the sound processor was initially activated after the standard healing phase. They also showed that the postoperative PTA_low_ decreased compared to the preoperative PTA_low_. With a deterioration in the PTA_low_ of 11.7 dB HL, the value is slightly lower than the data shown in the authors’ study, but in the study of Helbig et al., 2016 [16] a much higher number of cases was considered. As also observed in Helbig et al., 2016 [16], postoperative PTA_low_ in both EF and CG remained stable and showed no significant differences over time.

Both study groups showed comparable preservation of low-frequency residual hearing. A harmful impact of the early acoustic and electrical stimulation on the residual hearing can thus be excluded. Although the insertion of the electrode into the cochlear is rather traumatic [23] and intracochlear healing processes may take several weeks [2], early stimulation has shown no effect on postoperative residual low-frequency hearing, even over a longer period of time.

### Speech recognition

#### Freiburg monosyllabic and multisyllabic word score

Both study groups showed comparable development of multisyllabic and monosyllabic word score within the first year after CI surgery with a significant improvement in postoperative speech recognition at the 12-month interval (EF/CG 91.1/94.0% multisyllables, EF/CG 60.7/53.8% monosyllables) compared to preoperative results (EF/CG 70.0/69.5% multisyllables, EF/CG 13.2/22.35% monosyllables). The postoperative results of the Freiburg monosyllabic word score of both study groups are comparable to the results of other studies with similar age groups (50–60 years), in which an average speech recognition rate between 45 and 63% was described [17, 33–35]. Compared to preoperative results, the EF group showed an earlier significant improvement in postoperative speech recognition (after first fitting interval) than the CG (at 3-month interval). However, with an average of 27 years, it has to be taken into account that the CG had a substantial longer duration of hearing loss than the EF (17.5 years). Likewise, there was also a difference between EF (14.5 years) and CG (25.6 years) in mean hearing aid experience. It is known that the duration of hearing loss is related to the development of CI provided speech recognition. Clinical data showed that with increasing duration of hearing loss, the amount of improvement in speech recognition decreases [8, 17, 36]. In most patients with long-term partial deafness, the CI provided new auditory sensation cannot contribute immediately to improve speech recognition. It is assumed that the electrical stimuli are initially perceived as separate auditory objects and are not interpreted as belonging to corresponding speech signals [5]. When interpreting speech recognition results, the small number of cases as well as the large test/re-test variation of the results of the Freiburg monosyllabic word test must also be taken into account [37].

#### Oldenburg sentence test

In the OlSa in quiet as well as in the OlSa in noise comparable results in speech recognition were found in both study groups. At the 12-month interval there was no significant SRT difference between EF and CG. However, only a limited number of data sets were available for the OlSa both in quiet and in noise.

A precondition for EAS/hybrid use of the CI system is a successful postoperative preservation of residual low-frequency hearing [5, 19]. In both study groups postoperative low-frequency hearing could be preserved sufficiently in most cases. The EF patients were able to use the EAS/hybrid function to the same extent as the CG. In both groups, the EAS/hybrid function tended to be activated less frequently during the initial fitting (EF 80.0%/CG 78.3%) than during the 3-month interval (EF 90.0%/CG 86.7%). Due to postoperative wound swelling of the ear canal in some cases, the earmold (or dome) could not yet be worn (EF *n* = 6, 20%/CG *n* = 5, 21.7%). If sufficient residual low-frequency hearing was present, an EAS/hybrid map with corresponding cut-off frequency was usually created during initial device activation, but the acoustic part remained deactivated. In this time period, the patient used electrical stimulation only. The acoustic component of the EAS/hybrid system was activated at an additional appointment about four weeks after completion the initial fitting interval.

### Limitation of the study

A potential limitation of the study is that the results reported here likely depended on patient selection, which was not randomized. The patients chose freely between early fitting and standard fitting procedure. Subjects in the EF group might have had higher self-motivation toward CI than subjects in the CG. This notion seems to be supported by shorter duration of hearing loss and hearing aid experience. Therefore, a possible self-selected bias of the results cannot be excluded. Furthermore, due to complete case approach of the data, the consecutive limitation of the number of data sets is detrimental for the detection of significant effects.

## Conclusion

Early fitting of the sound processor had no negative effect on the preservation of the postoperative low-frequency residual hearing. In both EF and CG, long term preservation of the low-frequency hearing was possible. Both groups showed a comparable development of EAS/hybrid CI aided speech recognition within the first year after CI surgery. Due to the sufficient long term preservation of the residual low-frequency hearing, EF patients could benefit to the same extent from EAS/hybrid usage as patients who were fitted after the standard healing phase. The results of this study showed that early device activation is possible in EAS/hybrid CI patients with sufficient residual low-frequency hearing.

## Data Availability

Data available on request from the authors.

## References

[1] Hoppe U, Liebscher T, Hornung J (2017). Anpassung von Cochleaimplantatsystemen. HNO.

[2] Lenarz T (2017). Cochlear implant—state of the art. Laryngo-Rhino-Otol.

[3] Sun C-H, Chang C-J, Hsu C-J (2019). Feasibility of early activation after cochlear implantation. Clin Otolaryngol.

[4] Rader T (2015). Sprachverstehen mit elektrisch-akustischer stimulation: vergleich mit bilateral versorgten cochleaimplantatträgern in verschiedenen störgeräuschumgebungen. HNO.

[5] Baumann U, Helbig S (2009). Hören mit kombinierter elektrischer und akustischer stimulation. HNO.

[6] Sprinzl GM, Schoerg P, Edlinger SH (2020). Long-term hearing preservation in electric acoustic cochlear implant candidates. Otol Neurotol.

[7] Hagr A, Garadat SN, Al-Momani M (2015). Feasibility of one-day activation in cochlear implant recipients. Int J Audiol.

[8] Zeh R, Baumann U (2015). Stationäre Rehabilitationsmaßnahmen bei erwachsenen CI-Trägern: HNO. HNO.

[9] Prager JD, Neidich MJ, Perkins JN (2012). Minimal access and standard cochlear implantation: a comparative study. Int J Ped ORL.

[10] Stolle SRO, Groß S, Lenarz T (2014). Postoperative früh- und spätkomplikationen bei kindern und erwachsenen nach ci-implantation. Laryngo-Rhino-Otol.

[11] Stratigouleas ED, Perry BP, King SM (2006). Complication rate of minimally invasive cochlear implantation. Otolaryngol Head Neck Surg.

[12] Günther S, Baumann U, Stöver T (2018). Early fitting in cochlear implantation: benefits and limits. Otol Neurotol.

[13] Bruschke S, Baumann U, Stöver T (2021). Long-term follow-up of early cochlear implant device activation. Audiol Neurotol.

[14] Batuk MO, Yarali M, Cinar BC (2020). Is early cochlear implant device activation safe for all on-the-ear and off-the-ear sound processors?. Audiol Neurotol.

[15] Marsella P, Scorpecci A, Pacifico C (2014). Safety and functional results of early cochlear implant switch-on in children. Otol Neurotol.

[16] Helbig S, Adel Y, Rader T (2016). Long-term hearing preservation outcomes after cochlear implantation for electric-acoustic stimulation. Otol Neurotol.

[17] Hey M, Neben N, Stöver T (2020). Outcomes for a clinically representative cohort of hearing-impaired adults using the Nucleus^®^ CI532 cochlear implant. Eur Arch Otorhinolaryngol.

[18] Adunka O, Gstoettner W, Hambek M (2004). Preservation of basal inner ear structures in cochlear implantation. ORL.

[19] Kiefer J, Pok M, Adunka O (2005). Combined electric and acoustic stimulation of the auditory system: results of a clinical study. Audiol Neurotol.

[20] Cv I, Baumann U, Kiefer J (2011). Electric-acoustic stimulation of the auditory system: a review of the first decade. Audiol Neurotol.

[21] Cv I, Kiefer J, Tillein J (1999). Electric-acoustic stimulation of the auditory system—new technology for severe hearing loss. ORL.

[22] Adunka OF, Dillon MT, Adunka MC (2013). Hearing preservation and speech perception outcomes with electric-acoustic stimulation after 12 months of listening experience. Laryngoscope.

[23] Bas E, Dinh CT, Garnham C (2012). Conservation of hearing and protection of hair cells in cochlear implant patients’ with residual hearing. Anat Rec (Hoboken).

[24] Nadol JB, O’Malley JT, Burgess BJ (2014). Cellular immunologic responses to cochlear implantation in the human. Hear Res.

[25] German audiological society ADANO (2014) 017/010 - S1- Leitlinie Hörsturz: Hörsturz (Akuter idiopathischer sensorineuraler Hörverlust)

[26] Wagener K, Kühnel V, Kollmeier B (1999). Entwicklung und evaluation eines Satztests für die deutsche Sprache I: design des oldenburger satztests. Z Audiol.

[27] Bernhard N, Gauger U, Romo Ventura E (2021). Duration of deafness impacts auditory performance after cochlear implantation: a meta-analysis. Laryngosc Investig Otolaryngol.

[28] Suhling M-C, Majdani O, Salcher R (2016). The impact of electrode array length on hearing preservation in cochlear implantation. Otol Neurotol.

[29] Ramos-Macías A, Borkoski-Barreiro SA, Falcón-González JC (2017). Hearing preservation with the slim modiolar electrode nucleus CI532^®^ cochlear implant: a preliminary experience. AUD.

[30] Gautschi-Mills K, Khoza-Shangase K, Pillay D (2019). Preservation of residual hearing after cochlear implant surgery: an exploration of residual hearing function in a group of recipients at cochlear implant units. Braz J Otorhinolaryngol.

[31] Gstoettner W, Helbig S, Settevendemie C (2009). A new electrode for residual hearing preservation in cochlear implantation: first clinical results. Acta Otolaryngol.

[32] Hoskison E, Mitchell S, Coulson C (2017). Systematic review: Radiological and histological evidence of cochlear implant insertion trauma in adult patients. Cochlear Implants Int.

[33] Lenarz M, Sönmez H, Joseph G (2012). Effect of gender on the hearing performance of adult cochlear implant patients. Laryngoscope.

[34] Hast A, Schlücker L, Digeser F (2015). Speech perception of elderly cochlear implant users under different noise conditions. Otol Neurotol.

[35] Haumann S, Hohmann V, Meis M (2012). Indication criteria for cochlear implants and hearing aids: impact of audiological and non-audiological findings. Audiol Res.

[36] Cohen SM, Svirsky MA (2019). Duration of unilateral auditory deprivation is associated with reduced speech perception after cochlear implantation: a single-sided deafness study. Cochlear Implants Int.

[37] Winkler A, Holube I (2016). Test-retest-reliabilität des freiburger. HNO.

